# *Caesalpinia ferrea C. Mart.* (Fabaceae) Phytochemistry, Ethnobotany, and Bioactivities: A Review

**DOI:** 10.3390/molecules25173831

**Published:** 2020-08-23

**Authors:** Nair Silva Macêdo, Zildene de Sousa Silveira, Antonio Henrique Bezerra, José Galberto Martins da Costa, Henrique Douglas Melo Coutinho, Barbara Romano, Raffaele Capasso, Francisco Assis Bezerra da Cunha, Márcia Vanusa da Silva

**Affiliations:** 1 Laboratory of Semi-Arid Bioprospecting (LABSEMA), Regional University of Cariri—URCA, Crato 63105-000, CE, Brazil; naiirmacedo@gmail.com (N.S.M.); zildenesousa15@gmail.com (Z.d.S.S.); henriquebezerra.urca@gmail.com (A.H.B.); 2 Graduate Program in Biological Sciences—PPGCB; Federal University of Pernambuco—UFPE, Recife 50670-901, PE, Brazil; marcia.vanusa@ufpe.br; 3 Natural Products Research Laboratory, Regional University of Cariri—URCA, Crato 63105-000, CE, Brazil; galberto.martins@gmail.com; 4 Laboratory of Microbiology and Molecular Biology (LMBM), Regional University of Cariri—URCA, Crato 63105-000, CE, Brazil; hdmcoutinho@gmail.com; 5 Department of Pharmacy, University of Naples Federico II, 80100 Naples, Italy; barbara.romano@unina.it; 6 Department of Agricultural Sciences, University of Naples Federico II, 80055 Portici, Italy

**Keywords:** *Caesalpinia ferrea*, ethnoknowledge, bioactivities, phytochemicals, HPLC

## Abstract

*Caesalpinia ferrea C. Mart.*, popularly known as “Jucá” or “Pau-ferro”, belongs to the Fabaceae family, and is classified as a native and endemic species in Brazil. Numerous studies that portray its ethnobotany, chemical composition, and biological activities exist in the literature. The present study aimed to systematically review publications addressing the botanical aspects, uses in popular medicine, phytochemical composition, and bioactivities of *C. ferrea*. The searches focused on publications from 2015 to March 2020 using the Scopus, Periódicos Capes, PubMed, Google Scholar, and ScienceDirect databases. The leaves, fruits, seeds, and bark from *C. ferrea* are used in popular medicine to treat disorders affecting several systems, including the circulatory, immune, cardiovascular, digestive, respiratory, genitourinary, musculoskeletal, and conjunctive systems. The most commonly found chemical classes in phytochemical studies are flavonoids, polyphenols, terpenoids, tannins, saponins, steroids, and other phenolic compounds. The biological properties of the extracts and isolated compounds of *C. ferrea* most cited in the literature were antibacterial, antifungal, antioxidant, antiproliferative, anti-inflammatory, and healing potential. However, further studies are still needed to clarify a link between its traditional uses, the active compounds, and the reported pharmacological activities, as well as detailed research to determine the toxicological profile of *C. ferrea*.

## 1. Introduction

*Caesalpinia ferrea C. Mart*, formerly known as *Libidibia ferrea*, is a native and endemic species of Brazil, belonging to the Fabaceae family, popularly known as “Jucá” or “Pau-ferro”, and with several phytogeographic domain distributions, including the Cerrado, Caatinga, and Atlantic Forest [1].

Several parts from this species, such as the bark, leaves, fruits, and seeds, have been widely used by human beings in popular medicine through teas, decoctions, infusions, syrup, and macerations for countless therapeutic purposes, including: cicatrizing, anti-inflammatory, homeostatic, antiseptic, respiratory disorders, rheumatism, gastritis, among other purposes [2,3,4,5].

Therefore, several studies have investigated the phytochemicals and bioactivities attributed to *C. ferrea*, where, in terms of its chemical composition, studies have shown a diversity of chemical classes and compounds in the extracts from different *C. ferrea* organs. Flavonoids, organic acids, saponins, coumarins, phenols, and tannins are among the secondary metabolite chemical classes found in its extracts, while the phenolic acids, ellagic acid, and gallic acid, are the most commonly found compounds [6,7,8].

*C. ferrea* leaves, fruits, pods, and bark have been reported in the literature to have antibacterial, antifungal, anti-inflammatory, antioxidant, antidiabetic, and antiulcerogenic properties [9,10,11,12,13,14].

In the literature, there is a review about the biological properties of *C. ferrea* [15]; however, this review aimed to list the scientific information, covering five years, about the botanical aspects, traditional uses, phytochemistry, bioactivities, and the toxicity of *C. ferrea*, as well as discuss perspectives for new researches.

## 2. Results and Discussion

Following the database searches, 4069 studies were counted. Once the established inclusion and exclusion criteria were implemented, 87 articles were selected and analyzed for data extraction and result interpretation. It is worth noting that some publications analyzed both the chemical composition of the extract, as well as its biological effects.

### 2.1. Botanical Characterization

*Caesalpinia ferrea* is characterized for presenting an arboreal habit, with a height ranging from 10 to 15 m, alternating leaves and composed with alternating oval shaped leaflets with a hydrophobic character [16,17,18]. Inflorescences have flowers with yellow petals, an obovate shape and reddish spots [17,19]. The flowering period starts at the end of November and extends to the month of January, while the fruit ripening period comprises the months from July to August [20].

The fruits are flattened pods, which when immature are a green color and when ripe are a brown color, with this behavior being repeated with the seeds [19]. Table 1 represents the summary of the *C. ferrea* botanical characteristics. Its seeds are a determinant for the diffusion of the species; however, the seeds present dormancy caused by the impermeability of the tissue integument [21].

According to a study performed to evaluate the viability of *C. ferrea* seeds using the tetrazolium test, viable seeds had the following characteristics: bright light pink color, tissues with a normal and firm appearance, an intense red embryonic axis that did not reach the central cylinder, less than 50% of cotyledons were discolored or had necrotic regions, which did not interfere with the embryonic axis attachment area [22].

*C. ferrea* is a water demanding species for its growth, since when subjected to hydric stress conditions a relevant reduction in its height and leaf number was observed, indicating that water supply restrictions impaired the development of its morphological and physiological characteristics [23].

### 2.2. Ethnobotany

The ethnobotanical studies were selected according to the data provided for the plant part that was used, including preparation methods or the uses and therapeutic indications of *C. ferrea*, or its synonym *C. ferrea*. Table 2 summarizes the 19 articles addressing the medicinal uses of *C. ferrea*.

Ethnobotanical researches are important to understand the use of this plant by traditional communities and the general population for medicinal purposes and to maintain the folk culture. The results of ethnobotanical studies contribute with the association between traditional and modern knowledge, being an important tool to the investigation of the biological properties of medicinal plants.

Traditionally, *Caesalpinia ferrea* is used by traditional communities in northern and northeastern regions of Brazil to medicinal purposes [27,32,37,43,45,46,47]. The barks of *C. ferrea* are used by the native communities Cunuri, Tapira Ponta, Ilha das Flores, Curicuriari, and São Jorge, and riverside communities and rubber collectors located in the Amazon region, to combat malaria symptoms [25,32].

Given the analysis of the articles, the leaves were indicated as a vermifuge and for the treatment of infections and general inflammation, both of which used tea as a preparation method [5,24]. The bark from *C. ferrea* is one of the most commonly used organs in traditional medicine through the process of decoction, tea preparations, syrups and bottles for the treatment of various conditions which includes: flus, coughs, kidney and liver inflammation, anxiolytic, rheumatisms, diabetes, hemorrhages, inflammations, infections, and general pains [26,28,32,33,38,39].

The traditional use of the bark alongside other plant parts such as the bark, roots, and leaves, especially its fruits and seeds, has also been reported in the literature for various therapeutic indications, namely: syphilis, cancer, as a depurative, diabetes, gastritis, stomach pain, rheumatism, sexual impotence, cicatrizing, bone fractures, headaches, respiratory system disorders, fever, diarrhea, kidney problems, anxiolytic, vision problems, anti-inflammatory, analgesic, hematomas, anemia, colic, and shaking [27,31,34,40,42,45,46].

After the bark, the fruits are the most commonly used plant parts in the form of teas, bottled and macerated, for the treatment of diarrhea, liver, and kidney problems, pain (throat, legs, spine, and tooth), uterine inflammation, anemia, gastritis and urinary infection, as well as cicatrizing [29,35,41,43,44,47]. The seed tea is used to treat flus and coughs, and when immersed in water it is used as a cicatrizing agent [3].

### 2.3. Phytochemical Aspects

**Figure 1:**
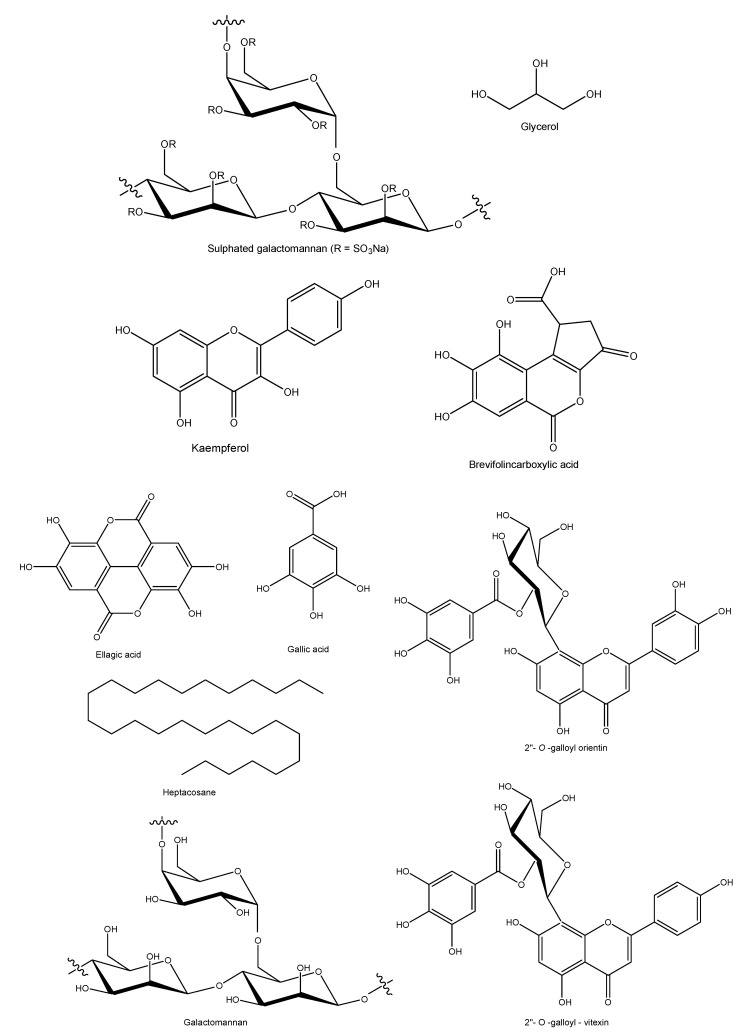
Chemical structures of the main compounds from *C. ferrea.* Ellagic acid: C_14_H_6_O_8_ and MW: 302.19 g/mol; Gallic acid: C_7_H_6_O_5_ and MW: 170.12 g/mol; Heptacosan: C_27_H_56_O and MW: 396.7 g/mol; Galactomannan: C_18_H_32_O_16_ and MW: 504.4g/mol; Kaempferol: C_15_H_10_O_6_ and MW: 286.24 g/mol; 2”-*O*-Galloylorientin: C_28_H_24_O_15_ and MW: 600.5 g/mol; 2”-*O*-Galloylvitexin: C_28_H_24_O_14_ and MW: 584.5 g/mol; Glycerol: C_3_H_8_O_3_ and MW: 92.09 g/mol.

Extracts from *C. ferrea* leaves, seeds, pods, and basts have been widely studied for having several secondary metabolites such as flavonoids, polyphenols, terpenoids, tannins, saponins, steroids, and other phenolic compounds that present a variety of bioactivities. In this review, 23 articles that investigated the phytochemicals in *C. ferrea* extracts were found (Table 3). Figure 1 represents the main phytocompounds from *C. ferrea*.

#### 2.3.1. Leaves

The qualitative phytochemical investigation of *C. ferrea* leaf extracts showed the presence of several chemical classes such as flavonoids, tannins, alkaloids, cinnamic derivatives, terpenes, saponins, organic acids, reducing sugars, steroids, triterpenoids, phenols, glycosides, phenolic compounds, and carbohydrates [8,9,57].

Gallic acid, brevifolin carboxylic acid, ellagic acid, brevifolin, tellimagrandin-I, 2”-*O*-galloylvitexin, vitexin, 2”-*O*-galloylorientin, orientin, isovitexin 2”-*O*-β- [xylopyranosyl-(1”” --- 2”’)-*O*-β-xylopyranosyl], isovitexin, orientin 2”-*O*-β- [xylopyranosyl-(1”” --- 2”’)-*O*-β-xylopyranosyl] were identified in the phytochemical composition of the leaf hydroethanolic extract using HPLC and NMR 1 and 2D techniques [49,50].

The leaves were subjected to two treatments, one with water at 100 °C and the other with water at 25 °C. After a HPLC phytochemical analysis of these extracts, gallic acid, caffeic acid, epicatechin, quercetin and luteolin were identified in both extracts, with ellagic acid and catechin also being identified in the hot extract (100 °C) [48]. Meanwhile, GC-MS analyzes of the cyclohexanic extract revealed the presence of n-dodecanal, octacosane, docosane, pentadecane, and heptacosane [8].

#### 2.3.2. Bark

The High-Performance Liquid Chromatography with Diode Array Detection (HPLC-DAD) analysis of the *C. ferrea* aqueous extract showed the presence of gallic, caffeic, and ellagic acids, catechin, epicatechin, and quercetin [47]. Whereas qualitative phytochemical analyzes of extracts with different solvent bases (ethanol, methanol, ethyl acetate and NaOH) presented different chemical classes such as flavonoids, tannins, saponins, steroids, terpenoids, coumarins, carbohydrates and proteins [6,51,53,58].

The phytochemical profile of the stem bark hydromethanolic extract was traced using the LC-MS/MS technique, detecting the presence of 15 compounds: quinolinic acid, gallic acid, 2-(2-ethyl-3-hydroxy-6-propionylcyclo-hexyl) acetic acid, ellagic acid, 12-oxo-phytodienoic acid, catechin, epicatechin, chlorogenic acid, rutin, taxifolin, myricetin, quercetin, kaempferol, apigenin, and isorhamnetin [57]. The chemical compounds ellagic and gallic acids were also found by HPLC in the aqueous and hydromethanolic extract [59,60].

#### 2.3.3. Fruits or Pods

The phytochemical analyzes of the aqueous and hydroalcoholic fruit extracts identified the presence of ellagic acid and gallic acid with the HPLC technique [13,55,61]. Meanwhile, qualitative chemical analyzes of the hydroalcoholic fruit extract showed the presence of seven chemical classes: saponins, organic acids, reducing sugars, phenols, tannins, sesquiterpene lactones, and anthraquinones [56].

LC-HRMS/MS analysis of the hydromethanolic fruit extract revealed the presence of phytochemicals such as gallic acid, galloyl-glucose ester, gallic acid methyl glycoside, hexose, di-*O*-galloyl-d-hexose, corilagin, ellagic acid, eriodictyol-*O*-hexoside and naringenin-*O*-hexoside [1].

Characterization of the pod hydroalcoholic extract chemical constituents revealed the presence of polyphenols (7.3%) and HPLC-MS chromatographic analyzes revealed the presence of nine compounds: galiliquinoic acid, galloyl-HHDP-hex, brevifolin carboxylic acid, valonium dilactone acid, gallic acid derivatives, ellagic acid derivatives (hex-ellagic acid), ellagic acid, and dihydroisovaltrate [12,14].

The LC-MS/MS phenolic composition investigation of the hydroethanolic pod extract showed the presence of gluconic acid, gallic acid, caffeic acid, 2-(2-ethyl-3-hydroxy-6-propionylcyclohexyl) acetic acid, ellagic acid, 12-oxo-phytodienoic acid, catechin, epicatechin, chlorogenic acid, rutin, taxifolin, myricetin, quercetin, kaempferol, apigenin, and isorhamnetin [55].

Phytochemical studies of the hydroalcoholic, chloroformic, n-hexane, and ethyl acetate extracts from *C. ferrea* pods showed the presence of numerous compounds such as glycerol, d-fructose, myo-inositol, chemical acid, glucopyranose, glucose, 1,2-benzenedicarboxylic acid, oxalic acid, butanedioic acid, pyrotartaric acid, pentanoic acid, malic acid, pentanedioic acid, arabinonic acid, octanedioic acid, azelaic acid, d-galactopyranosyl, benzoic acid, alpha-d-glucopyranose, palmitic acid, stearic acid, 2-bromosbacic acid, tetracosanoic acid, n-valeric acid, alpha hydroxyisobutyric acid, caproic acid, heptanoic acid, octanoic acid, maleic acid, pyrotartaric acid, pelargonic acid, pimelic acid, tetradecanoic acid, suberic acid, myristic acid, D-mannose, n-pentadecanoic acid, palmitic acid, cholesterol, 2-bromosbacic acid, monopalmitin, docosanoic acid, N-dodecanol, myristic acid, methyl palmitate, palmitic acid, methyl oil, methyl stearate, vapor acid, methyl arachidate, arachidonic acid, methyl benzoate, methyl lignocerate, tetracosanoic acid, nonacosane, octacosanol, and campesterol [56].

#### 2.3.4. Seeds and Roots

The seed hydroalcoholic extract presented ellagic acid as its main phytochemical constituent and Thin-Layer Chromatography (TLC) qualitative chemical analyzes showed the presence of fatty acids, coumarins, as well as total and hydrolyzable tannins [59,62]. The root aqueous extract made with water at different temperatures (100 and 25 °C) presented the following constituents in common: gallic and ellagic acid, epicatechin, quercetin and luteolin, while the hot extract (100 °C) also presented catechin in its composition [48].

#### 2.3.5. Compostos De Valor Quimiotaxônomico

The species of the subfamily Caesalpiniaceae present hydrophilic polysaccharides such as galactomannans with a the mannose/galactose ratio ranging from 2.5:1 to 4.3:1 [63]. In *C. ferrea*, the presence of these polysaccharides was detected by different authors, and these biomolecules are chemotaxonomic markers used to the analysis and identification of these species [64,65]. Other chemical compounds useful as chemotaxonomic markers of Caesalpiniaceae are flavonoids, terpenoids, and isoflavonoids [12,14,48,66].

### 2.4. Bioactivities

A total of 57 articles investigating the bioactivity of the extracts and compounds isolated from *C. ferrea* were found. Table 4 represents the summary of the extracts’ bioactivities and Table 5 corresponds to those of the isolated compounds.

#### 2.4.1. Antimicrobial Activity

The antibacterial activity of the *C. ferrea* pod hydromethanolic extract was evaluated against oral bacteria that are commonly associated with bad breath, where a Minimum Inhibitory Concentration (MIC) of 50 µg/mL and a Minimum Bactericidal Concentration (MBC) >50 µg/mL were obtained for *Parvimonas micra*, whereas the *Porphyromonas gingivalis* microorganism obtained a MIC of 120 µg/mL and a MBC of 130 µg/mL [14]. Moreover, the pod hydroalcoholic extract presented an antibacterial activity against *Helicobacter pylori* with a MIC and MBC of 512 µg/mL [12].

Ethanol extracts from *C. ferrea* bark and pods show MIC values of 1024 µg/mL to *Staphylococcus aureus* (ATCC 25293), *Escherichia coli* (ATCC 25922) and *Pseudomonas aeruginosa* (ATCC 9027) strains, as well as multi-drug resistant *S. aureus* (SA10), *E. coli* (EC06), and *P. aeruginosa* (PA 32) strains [70].

The bark hydroalcoholic extract presented an antimicrobial potential with species from the *Staphylococcus* spp., obtaining inhibition halo sizes of 61.1%, 27.78%, and 5.56% for the crude extract at the 70% and 50% concentrations, respectively [70]. The aforementioned extract also presented an antimicrobial activity against *S. aureus* ATCC 10390, *P. aeruginosa* ATCC 9721, and *E. coli* ATCC 25,922 strains with MIC values of 0.39, 0.79, and 0.19 mg·mL^−1^, respectively [67].

The antibacterial activity of *C. ferrea* leaf extracts was evaluated against several bacterial strains: *Bacillus subtilis*, *E. coli*, *Proteus vulgaris*, *P. aeruginosa*, *S. aureus*. The *C. ferrea* methanolic extract showed a MIC of 6.25, 12.5, 25, 3.12, 3.12, 12.5, 0.78, 3.12, and 12.5 mg/mL, respectively, while the cyclohexane extract obtained MIC values of 0.039, 0.039, 0.039, 0.39, 0.078, 0.312, 0.039, 0.078, and 0.039 mg/mL, respectively. The chloroform extract presented MIC values of 1.56, 6.25, 12.5, 3.12, 0.78, 6.25, 0.78, 12.5, and 12.5 mg/mL, respectively. The ethyl acetate extract showed MIC values of 0.78, 6.25, 12.5, 3.12, 1.56, 6.25, 0.39, 3.12, and 3.12 mg/mL, respectively [8].

The *C. ferrea* leaf and fruit aqueous extracts presented a 70% growth inhibition potential for *Ralstonia solanacearum* [69]. Meanwhile the glycolic leaf extract presented an antimicrobial potential against the following strains: *Streptococcus mutans* ATCC 25175, *Streptococcus mitis* ATCC 9811, *Streptococcus sanguis* ATCC10556, *Streptococcus sobrinus* ATCC 27,609, and *Lactobacillus casei* ATCC 7469 [72].

The *C. ferrea* fruit hydroalcoholic extract presented antimicrobial potential against the following strains: *S. aureus*, *E. coli*, *Klebsiella pneumoniae,* and *P. aeruginosa* with inhibition halo averages of 18, 12, 10 and 11 mm, respectively [68]. The *C. ferrea* pod ethanolic extract also presented antimicrobial activity against Gram-positive and Gram-negative strains with MIC values ranging from 50 to 125 μg/mL [73]. The glycolic leaf extract on the other hand exhibited an average inhibition halo of 0.97 cm over *S. aureus* ATCC 6538 [57].

The *C. ferrea* fruit aqueous extract presented antibiofilm activity for biofilms formed by bacteria from the Proteobacteria and Bacteroidetes phylum, inhibiting their growth by 82% at a concentration of 4 mg·mL^−1^ [74].

#### 2.4.2. Anti-Halitosis Activity

Volatile sulfuric compounds are produced by bacteria present in the oral cavity, with these compounds being responsible for unpleasant breath odors. Tests using a salivary sediment model have shown the *C. ferrea* pod hydromethanolic extract inhibited the formation of these odors, reducing the concentration of volatile sulfuric compounds associated with halitosis [14].

#### 2.4.3. Antifungal Activity

The *C. ferrea* seed hydroethanolic extract presented antifungal activity against *Candida albicans* ATCC 10231, *C. glabrata* CCT 0728, *C. krusei* CCT 1517 and *C. guilliermondii* CCT 1890 strains with MIC values that varied between 4.8–78 µg/mL [11,75].

Extracts from the *C. ferrea* stem bark using three different solvents (water, ethanol, and acetone) exhibited antifungal activity over dermatophyte fungi species, presenting the same MIC value of 62.5 μg/mL for *Trichophyton rubrum* ATCC 28,189 and 31.3 μg/mL for *Trichophyton mentagrophytes* ATCC 11481, with the author classifying MIC values ≤ 75.0 μg/mL as having an effective antifungal activity. The stem bark aqueous extract also showed antifungal activity over clinical isolates (*T. rubrum* and *T. mentagrophytes*) with a MIC_50_ value of 31.3 μg/mL and MIC_90_ value of 62.5 μg/mL for both species, where MIC_50_ and MIC_90_ refer to the concentration (μg/mL) of the extract that inhibited growth of all isolates by 50% and 90%, respectively [78].

Tests using the *C. ferrea* leaf ethanolic extract as an alternative control for Alternaria brown spots, caused by the fungus *Alternaria alternata* in ‘Dancy’ mandarin fruits, showed the 500 μg/mL concentration presented a 52.0% disease severity reduction [93]. The aqueous and ethanolic extracts also showed 96.49% and 99.12% disease severity reduction in ‘Ponkan’ tangerine seedling leaves, respectively, at the 1 mg/mL concentration [94].

The *C. ferrea* leaf alcoholic extract demonstrated an antifungal potential over *Colletotrichum* sp. in *Sideroxylon obtusifolium* (“quixaba”) seeds, since all seeds treated with the extract did not present symptomatic seedlings percentages or pathogen transmission rates [77]. The aqueous extract also demonstrated an antifungal activity over *Colletotrichum* sp. in *S. obtusifolium* seeds, since pathogen incidence decreased by up to 96% at the 0.075 mg·mL^−1^ concentration [76]. *C. ferrea* extracts also showed antifungal activity over *Lasiodiplodia theobromae*, inhibiting mycelial growth by 85.6 and 78.9% at 30% and 20% concentrations, respectively [79].

#### 2.4.4. Anti-Inflammatory Activity

The *C. ferrea* leaf aqueous extract decreased leukocyte accumulation (76 ± 2%) and myeloperoxidase levels (85 ± 7%) in the articular fluid of rats at 100, 200, and 300 mg/kg doses when compared to the zymosan control group, a substance used to stimulate intra-articular inflammation. In addition, the extract significantly reduced inflammatory cytokine levels such as beta interleukins (IL-1β) and tumor necrosis factor alpha (TNF-α) in the articular tissue of rats treated with 200 and 300 mg/kg doses [7]. The fruit aqueous and hydroalcoholic extracts (20–80%) also showed an anti-inflammatory activity at all tested doses (50, 100, and 200 mg/kg), decreasing the migration of inflammatory cells and myeloperoxidase activity levels [13].

#### 2.4.5. Antioxidant Activity

Generally, two parameters are used to assess the integrity of the body’s antioxidant defense: the first is the quantity of glutathione, which is responsible for promoting detoxification and free radical elimination, the second is malondialdehyde (MDA) content, which is characterized as a lipid peroxidation marker. The fruit and leafs aqueous and hydroalcoholic extracts of Pau-ferro showed antioxidant potential at all evaluated doses (50, 100, and 200 mg/kg), increasing total glutathione levels and decreasing MDA levels [7,13,54].

The *C. ferrea* leaf hydroethanolic extract and the compounds isolated from this extract (brevifolin carboxylic acid, 2”-*O*-Galloylvitexin and 2”-*O*-Galloylorientin) presented an antioxidant activity through its ability to absorb oxygen radicals in HaCaT keratinocytes, presenting effective dose values (ED_50_) of 12.5, 5, 3.8, and 1.9 µg/mL, respectively [50].

The *C. ferrea* leaf ethanolic extract demonstrated antioxidant potential, where an ED_50_ of 12.45 ± 2.86 µg/mL was obtained, exhibiting a marked activity in radical elimination in the 2,2-diphenyl-1-picrylhydrazil (DPPH) assay using male Sprague–Dawley rats as an experimental model [9]. Other positive results were also found using the same extract with an EC_50_ of 4.4 μg/mL (DPPH) and 2.5 μg/mL 2,2’-azino-bis (3-ethylbenzothiazoline) (ABTS) demonstrating high free radical scavenging activity [10,73].

The leaf hydroalcoholic extract also showed high antioxidant activity in the DPPH and ABTS free radical elimination assays with Half Maximal Inhibitory Concentration (IC_50_) values of 28.96 and 145.10 μg/mL, respectively [12]. Four pod extracts (chloroformic, n-hexane, hydroalcoholic, and ethyl acetate) were evaluated for their antioxidant activity; however, only two extracts presented free radical elimination activity, these being the hydroalcoholic extract, with IC_50_ values of 74.36 μg/mL for DPPH and ABTS IC_50_ of 9.76 μg/mL, and the ethyl acetate extract, with IC_50_ values of 116.10 and 29.13 μg_/_mL for DPPH and ABTS, respectively [56].

#### 2.4.6. Antileishmanial Activity

The fruit methanolic extract and leaf hexanic extract showed antileishmanial activity, with IC_50_ values of 15.04 and 53.09 μg·mL^−1^ against *Leishmania* (*L.*) *amazonensis* promastigotes. Meanwhile, the leaf and fruit methanol extracts showed an IC_50_ activity of 129.42 and 173.11 μg·mL^−1^ against *Leishmania* (*V.*) *guyanensis* promastigotes. The fruit methanol extract showed low in vitro toxicity on infected macrophages, and was thus selected for antileishmanial activity tests with intracellular infected macrophages, the results of which showed the 500 μg.mL^−1^ concentration inhibited 62% and 54% of *L. (V.) guyanensis* and *L. (L.) amazonensis* amastigotes survival, respectively [80].

#### 2.4.7. Antiproliferative and Apoptotic Effects

*C. ferrea* fruit ethanol extracts were evaluated for their antiproliferative effects on human colorectal cancer cells (HT-29), with a potential for inhibiting cancer cell proliferation being observed for extracts with 40, 60, and 80% ethanol, where the following results can be highlighted: 15–25% proliferation inhibition between 25 and 100 μg/mL doses of the 40% ethanolic extract, while the 60% ethanolic extract showed a 50% rate of proliferation inhibition at the 25 μg/mL dose, and the 80% ethanolic extract showed 43.7% inhibition at the 12.5 μg/mL dose, where all of results were observed in the first 24 h of the experiment. During this same period, the extracts did not present embryonic renal cell line (HEK-293) toxicity, these being tumor-free [54].

As for apoptotic effects, the fruit extract with 40% ethanol at a 25 μg/mL dose presented a high percentage of cells undergoing apoptosis (38.7%) in the HT-29 tumor line, while the number of cells undergoing apoptosis in HEK-293 non-tumor cell line did not differ statistically from the control [54].

#### 2.4.8. Anti-Wrinkle and Anti-Melanogenic Activity

The *C. ferrea* pod and bark ethanolic extract showed high elastase inhibitory activity, with 35.99% inhibition at 250 µg/mL for the bark extract and 19.6% for the pod extract. In terms of collagenase activity, the extracts did not show significant inhibitory potentials, whereas for hyaluronidase, the two extracts obtained better inhibitory potentials than the control [53].

The anti-melanogenic effect of the two extracts were analyzed in B16F10 cells (murine melanoma), where the cells were pre-treated with 3-isobutyl-1-methylxanthine (IBMX) and showed an increase in tyrosinase activity before receiving treatment with the extracts at the 25 μg/mL concentration for 48 h. After treatment with the extracts, significant reductions of 99.0 and 96.4% in tyrosinase activity were observed when treated with the bark and pod extracts, respectively [53]. Tests analyzing the photoprotective activity of the bark ethanolic extract demonstrated a sun protection factor (SPF) of 3.29 at the 0.100 mg/mL concentration [6].

#### 2.4.9. Anti-Hyperglycemic Activity

A galactomannan extracted from *C. ferrea* seeds demonstrated an anti-hyperglycemic activity when orally administered to streptozotocin-induced diabetic rats at a 10 mg/kg dose. During the first days of treatment, a reduction in blood glucose and blood triacylglycerol levels was observed, in addition to a boost in adipose tissue insulin sensitivity, contributing to the functional recovery of the tissue [65].

Oral administration of the *C. ferrea* leaf ethanolic extract also showed anti-hyperglycemic activity in streptozotocin-induced diabetic rats, reducing liver function levels, elevated serum glucose and a-amylase, while, in contrast, increasing serum insulin levels, total proteins and body weight [9].

#### 2.4.10. Antiviral Activity

Sulfated galactomannan extracted from *C. ferrea* exhibited 96% inhibition at a concentration of 25 g/mL against the dengue virus (DENV-2), as well as showing strong antioxidant activity in Vero cells infected with the dengue virus (DENV-2), with an IC_50_ of 0.94 μg/mL [100].

#### 2.4.11. Antinociceptive Activity

The *C. ferrea* fruit aqueous extract exhibited an analgesic activity in rats during the hot plate test at 100 and 200 mg/kg doses at 90 and 60–90 min, respectively [13].

#### 2.4.12. Antiulcerogenic Activity

The *C. ferrea* dry extract exhibited an antiulcerogenic activity in Wistar rats with lesions induced by absolute ethanol obtaining inhibition values of 46.36, 87.56, and 95.99% at 100, 200, and 400 mg/kg doses, respectively. In ulcers induced by acidified ethanol administration, the extract showed a protection of 59.12 and 96.61% in the group treated with 200 and 400 mg/kg doses. At the end of the tests used to evaluate the gastroprotective potential of the extract, Half Effective Maximum Dose (ED_50_) values of 113 and 185.7 mg/kg were obtained for the groups with ulcers induced by absolute ethanol and acidified ethanol, respectively. In addition, the 200 mg/kg dose decreased the area of chronic ulcers induced by acetic acid by 77.44% [12].

#### 2.4.13. Hypolipidemic Effects

The *C. ferrea* leaf hydroethanolic extract was evaluated for its in vitro hypolipidemic effect on the activity of HMG CoA-reductase, an enzyme responsible for cholesterol biosynthesis. The extract showed an enzymatic inhibitory activity of 86%, a result similar to that of the medication Lipantil used as the positive control. In in vivo tests with Wistar rats, the group treated with the extract showed significant reductions in total cholesterol (TC), low density lipoprotein cholesterol (LDL-C), triglycerides (TG) and total lipids of 53.08, 25.03, 48.84 and 23.28%, respectively, while HDL-C showed a significant increase of 158.71% compared to the untreated group, that is, the hypercholesterolemic group. Histopathological analyzes showed that rats treated with the extract had normal-looking livers and a very mild presence of congestion and degenerative changes [50].

#### 2.4.14. Toxicity

Pregnant rats treated with *C. ferrea* bark and seed extracts showed changes in some biochemical parameters, among which increases in creatinine levels in the maternal serum stood out when compared to the control. Additionally, the group of rats exposed to the seed extract presented amniotic fluid rich in glucose and aspartate aminotransferase and low levels of calcium, which as a result, the fetuses had shorter head and body section lengths when compared to the control, as well as exhibiting visceral and skeletal anomalies [59].

No behavioral changes were observed through toxicological tests using adult zebrafish (*Danio rerio*) as a model organism when these were exposed to the *C. ferrea* fruit ethanolic extract, however, histopathological analyzes of different tissues showed changes in their gills, intestines, and livers. In contrast, assays using 25, 50, and 125 mg/L concentrations of the extract showed embryonic lethality rates of 30, 33.3, and 10%, respectively, while higher concentrations (250 and 500 mg/L) triggered edema in the heart, yolk sac and scoliosis [1]. Meanwhile, the bark hydroalcoholic extract showed an LC_50_ of 822.6334 µg/mL in the toxicity evaluation assay over *Artemia salina* L., this being considered a low toxicity [81]. The evaluation of the *C. ferrea* leaf ethanolic extract toxicity demonstrated a non-toxic profile, since the rats subjected to the extract did not exhibit significant behavioral changes, neurological responses or mortality rates in any of the doses until the end of the assays [9]. Similar results were found in the acute toxicity evaluation of the fruit ethanol extract and the pod hydroalcoholic extract in Wistar rats, with a lethal dose (LD) greater than 2000 mg/kg for the pod extract [12,61].

#### 2.4.15. Genotoxicity

The genotoxic activity of the *C. ferrea* seed ethanol extract on *Astyanax* sp. erythrocytes was measured through the comet assay, where the tail length increased the level of DNA breaks in red cells by 2.5× when exposed to 5, 10, and 20 mg/L doses compared to the negative control; thus, demonstrating a genotoxic potential over *Astyanax* sp. erythrocytes, in addition, a clastogenic response following exposure to the 20 mg/L dose, as evidenced by a decrease in tail length [82].

#### 2.4.16. Cytotoxicity

The cytotoxic potential of the *C. ferrea* leaf hydroethanolic extract over human cancer cell lines (liver HepG2, breast MCF-7, colon HCT-116, larynx Hep2 and prostate PC3) was analyzed using the sulforhodamine B (SRB) assay. The results showed the extract presented cytotoxic activity over the five tumor cell lines analyzed with an IC_50_of 19.3 μg/mL for the HepG-2 liver cell line, this being considered the most efficient cytotoxic activity. As for the other lines, larynx Hep2, breast MCF-7, and colon HCT-116, IC_50_ values of 20, 21.8, and 24.47 μg/mL, respectively, were obtained, while a negative cytotoxic activity was observed with the PC3 prostate cell line [49].

The 2”-*O*-galloylvitexin compound was isolated from the *C. ferrea* leaf hydroethanolic extract and was analyzed for its cytotoxicity potential over the aforementioned strains using the same methodology, presenting similar results with more effective cytotoxic activity on the HepG-2 liver cell line with an IC_50_ of 18.5 μg/mL, followed by the HCT-116 colon, Hep2 larynx, and MCF-7 breast cell lines with IC_50_ values of 22.6, 24.2, and 28.4 μg/mL, respectively [49].

The cytotoxicity of the *C. ferrea* bark and pod ethanolic extracts were evaluated against B16F10 cells (murine melanoma) and normal human fibroblasts (NHF). The pod extract obtained an IC_50_ of 50.1 µg/mL for the B16F10 cells after the 48-h treatment period, while the bark extract showed a 47% viability percentage of B16F10 cells. Neither extract presented significant cytotoxicity in over NHF [53].

The fruit aqueous and hydroalcoholic extracts (20–80%) had no cytotoxic effect over mouse embryonic fibroblast (3T3) cell lines at any of the periods analyzed (24, 48, and 72 h) [13]. Tests using the *C. ferrea* seed hydromethanolic extract demonstrated toxicity in fibroblast cells (L929) when used at concentrations of 1000, 500, and 250 μg/mL, while concentrations below 250 μg/mL did not show cytotoxicity [75].

The cytotoxicity potential of galactomannan extracted from *C. ferrea* seeds was evaluated, in vitro, in human neutrophils through the lactate dehydrogenase (LDH) assay, where it is possible to detect cell death, such as necrosis. The results showed that galactomannan did not increase LDH activity at any of the analyzed concentrations (10–200 μg/mL) when compared to the negative control data [65]. Assays with sulfated galactomannans at concentrations of 25, 50, and 100 g/mL also did not show cytotoxicity in Vero cells [100].

The cytotoxic potential of the *C. ferrea* leaf hydroethanolic extract and its isolated compounds such as brevifolin carboxylic acid, 2”-*O*-Galloylvitexin and 2”-*O*-Galloylorientin were investigated in HaCaT keratinocytes by the neutral red uptake (NRU) test. The results from the cytotoxic activity showed IC_50_ values of 114.4, 124.9, 59.7, and 67.5 μg/mL for the extract, brevifolin carboxylic acid, 2”-*O*-Galloylvitexin and 2”-*O*-Galloylorientin, respectively [50].

The *C. ferrea* pod aqueous extract exhibited cytotoxicity over meristematic cells from *Allium cepa* roots, inhibiting cell division at concentrations of 1 g/500 mL and 1 g/1000 mL following 24 and 48 h of exposure. Moreover, the extract showed a cytoprotective effect at both concentrations in tests used to evaluate their cytoprotective potential in cells treated with paracetamol at a concentration of 0.008 mg/mL. In addition, the extract did not contribute to the antiproliferative activity caused by a mutagenic compound in meristematic cells from *A. cepa* roots [83].

#### 2.4.17. Cicatrizing Activity

Powder and ointment formulations developed from *C. ferrea* pods were evaluated for their cicatrizing potential in clean rabbit (*Oryctolagus cuniculus*) wounds, following three times a day administration. The results showed that animals treated with the 24% ointment showed a better lesion area percentage inhibition, while inefficiency at reducing the final cicatrizing period was observed with the other treatments [84].

*C. ferrea* bark extracts that are rich in polysaccharides at concentrations of 0.025, 0.05, 0.75, and 0.1% decreased the wound area and increased wound contraction in Wistar rats, in addition to reducing the infiltration of inflammatory mediators, such as TNF-α and IL-1β, contributing to the acceleration of the wound healing process, as evidenced by the presence of attenuated clinical signs (edema, hyperemia, exudate). Likewise, ulcers treated with the extract showed the formation of organized connective tissue and collagen deposition, as well as a layer of epithelial tissue protecting the granulation tissue [58].

Positive results were found for the wound cicatrizing process in Wistar rats using the *C. ferrea* pod powder, with a significant reduction in the lesion area occurring and the wounds drying with no exudate from the third day of treatment, exhibiting a regular, thick crust with the presence of mononuclear red blood cells and fibrin, in the form of a blood clot at the edges of the wound [85].

Cutaneous wound treatment in Wistar rats with the *C. ferrea* fruit ethanolic extract stimulated the formation of dark brown to black crusts over the wounds of treated animals at concentrations of 50 and 12.5%, where crust detachment was also observed during topical treatment days with the extract. In the group treated with the extract at a concentration of 12.5%, all animals exhibited a constituted epidermis, this being more efficient at skin wound treatment in rats than the 50% concentration [61].

Diabetic and non-diabetic Wistar rats with lesions induced by thermal contact were treated with the *C. ferrea* bark hydroalcoholic extract incorporated with bacterial nanocellulose membranes, where the non-diabetic rat group exhibited epithelialization after 14 days of treatment, while the remaining animals presented epithelialization after 21 days of treatment [67].

#### 2.4.18. Repellent Action

The repellent action of the *C. ferrea* fruit bark powder against fly species from the *Calliphoridae* family was analyzed using traps containing deteriorating bovine liver as an attractant for the flies. The treatments that had the powder at the 20 and 50% tested concentrations presented a higher repellency percentage of 97.5 and 100%, respectively [88].

#### 2.4.19. Insecticidal Activity

The insecticidal activity of the *C. ferrea* leaf hydroalcoholic extract was evaluated using *Aphis craccivora* (black aphid) nymphs, which demonstrated the 5% concentration showed insecticidal activity with 51.71% efficiency [90]. The insecticidal activity of the leaf and pod aqueous and methanolic extracts against *Dactylopius opuntiae* (“Cochonilha-do-carmin”) were also verified, with a 72.46–99.33% of adult female mortality being observed [91]. Results using the same extract and *D. opuntiae* demonstrated LC_50_ values ranging from 20–150 mg/mL for the nymphs and 43–50 mg/mL for the adults, while other tests using termites as a study model (*Nasutitermes corniger*) presented LC_50_ values ranging from 0.255–1.279 mg·mL^−1^ for the workers and 0.146–8.003 mg·mL^−1^ for the soldiers [89,92].

#### 2.4.20. Fertilizer

The *C. ferrea* leaf litter was used to evaluate its fertilizing potential against *Sorghum bicolor* L. (Sorgo) cultures, where following a period of 75 days the *C. ferrea* litter increased potassium, calcium, and magnesium soil content; however, it did not increase the dry matter production of the *S. bicolor* L. aerial part [95].

#### 2.4.21. Allelopathic Potential

The *C. ferrea* seed hydroalcoholic extract presented a 30% rate of abnormal melon seedlings (*Cucumis melo* L.) at the highest concentration (1%), where the seedlings had imperfect roots, including the absence of absorbent hair, a necrotic, dark, and hard apex, in addition to negative gravitropism. The leaf hydroalcoholic extract on the other hand contributes to the growth of the melon seedling aerial parts at the highest tested concentration (1%), while it negatively interfered with root growth compared to the control [96]. 

Decomposing *C. ferrea* leaves exhibited an allelopathic potential over *Vigna unguiculata* L. seedlings, affecting the length of the aerial part and root system, as well as the total seedling dry mass [97]. The hot leaf, bark, and root extracts exhibited allelopathic potential over *Calotropis procera*, preventing its germination, while *Cenchrus echinatus* germination was inhibited by the hot leaf extract [48].

#### 2.4.22. Biosorbent

Tests with a biosorbent produced from *C. ferrea* fruits used to remove methylene blue from aqueous media showed rapid kinetics coupled to good adsorption, demonstrating that the fruits can become an excellent lower cost alternative used for the removal of pollutants in wastewater [98]. Activated coals prepared from pod waste showed a removal percentage of up to 97.67% of the pharmaceutical captopril from aqueous media [99].

#### 2.4.23. Other Bioactivities

The *C. ferrea* stem bark tea presented an erosive potential on human third molars, with a pH value of 0.28 ± 0.05, this being considered close to the pH value that promotes tooth enamel demineralization, with 37.03% of enamel demineralization [87].

The pod hydroalcoholic extract showed an antimetastatic potential, since it decreased the migration of ACP02 cells (gastric adenocarcinoma) following 24 h of treatment, presenting a dose-response effect that increased from 50 µg/mL concentrations [56]. The *C. ferrea* seed aqueous extract did not have the potential to inhibit hemorrhages induced by *Bothrops jararaca* venom in Swiss mice [62].

Polysaccharide extracts from *C. ferrea* leaves, pods and barks showed an edematogenic effect in Wistar rats at 0.01–1 mg/kg doses, with these effects being inferior to those developed by the drugs used as control (carrageenan and dextran) [86].

## 3. Materials and Methods

The plant’s name was checked on the www.theplantlist.org/ (The List Plant) website to check for synonyms. For data collection, a comprehensive article search was performed using the Scopus, Periódicos Capes, PubMed, Google Scholar, and ScienceDirect databases, using the following descriptors: *Caesalpinia ferrea* and its synonym *Libidibia ferrea*. Studies published between 2015 and March 2020 were reviewed. Full-text articles were selected if the title, abstract, or keywords included the aforementioned descriptors.

Articles duplicated between the search engines, as well as review articles, were removed in the first step. The remaining articles were then selected based on their title, abstract, and keywords. Lastly, full articles were analyzed according to the following criteria (1): botanical aspects; (2): phytochemistry; (3): ethnobotanical uses; (4): bioactivities; (5): bioactivities of compounds isolated from *C. ferrea*. The chemical structures of the isolated secondary metabolites of this plant were drawn using the software ChemDraw Ultra 7.0.

## 4. Critical Analysis

Phytochemical investigations are fundamental for understand the chemical basis of the compounds from medicinal plants; however, many of these studies still use colorimetric techniques that have limitations. By this fact, is necessary the usage of metabolomic techniques to trace the phytochemical profiles. The phytocompounds of *C. ferrea* have a wide range of therapeutic properties, however only few compounds were isolated and evaluated by in vivo and in vitro assays, as evidenced in this review. The studies that evaluated pharmacological activities did not show results about the mechanisms of action of the crude extracts and the isolated compounds. By this fact, investigations to evaluate the toxicological in vivo effects are necessary to understand the pharmacokinetic and pharmacodynamic mechanisms of these products. Pharmacological studies have focused on the antibacterial, antioxidant, anti-inflammatory, antihyperglycemic and healing effects, demonstrating the ethnomedicinal uses of this plant. However, many traditional uses are not supported to experimental results, as the effects against malaria, anemia, and the calming properties of this plant.

## 5. Conclusions

In this work, we reported the ethnobotanical, phytochemical, and pharmacological aspects of *C. ferrea*. This medicinal plant is used in traditional practices to treat certain diseases and has interesting biological properties. Different pharmacological assays had demonstrated antibacterial, antioxidant, anti-inflammatory, anti-diabetic, and healing activities. However, these pharmacological investigations have focused mainly on the organic fractions of the crude extracts, with few attentions to the aqueous extracts, since that are the mainly used formulations. Another point is the necessity of more comprehensive clinical trials. Regarding the phytochemical analysis of this species, different compounds as flavonoids, polyphenols, terpenoids, tannins, saponins, steroids, and other phenolic compounds were reported. However, the reports about the pharmacological properties cited in this review have not demonstrated the mechanisms molecular of *C. ferrea* extracts. Therefore, it is necessary to develop new research to establish a link between traditional uses and pharmacological activities, mainly studies to determine the toxicological profile of *C. ferrea*.

## Figures and Tables

**Table 1:** Summary of the *C. ferrea* botanical characteristics.

Characteristics	Attributes	Citations
Habit	Arboreal	[17]
Height	10–15 m	[18]
Leaves	Alternating and composed	[16]
Flowers	Inflorescences with yellow petals	[17,19]
Fruits	Flattened pods	[19]
Seeds	Brown when ripe	[19]

**Table 2:** Traditional uses of *C. ferrea* for curing diseases.

Part Used	Method of Preparation or Use	Therapeutic Indication	Citation
Leaf	Tea	Vermifuge	[24]
Leaf, bark and fruit	Decoction, “lambedor”, maceration, medicinal wine	Asthma, bones pain, flu, kidney pain, cough, shaking	[25]
Bark	Decoction	Liver/bleeding	[26]
Pod, fruit, seed, and bark	Tanned in wine, tea, bath, macerated, cooked beaten with water	Anti-inflammatory and healing	[27]
Bark and fruit	Tea, “lambedor” and syrup	Flu, kidney inflammation and soothing	[28]
Fruit	“Lambedor”	Flu	[29]
Bark and seeds	Hurt the seed and soak it in the water	Pneumonia, anemia, diarrhea, colic and gastritis	[30]
Stem bark, fruit, and seed	Maceration	Anti-inflammatory, kidneys, bruises, back pain, healing, analgesic	[31]
Bark	Decoction	Malaria	[32]
Bark and root	Tea and bottles	Rheumatism and diabetes	[33]
Bark and fruit	Bottles	Anti-inflammatory	[34]
Fruit	Tea	Diarrhea, liver and healing	[35]
Stalk	Tea	Anti-inflammatory	[36]
Roots	Decoction	Hemorrhoids, inflammation of the eyes and injuries	[37]
Whole shell	Immersed in water	Hemorrhage, anti-inflammatory, infection and pain	[38]
Dry bark	Decoction	Back pain	[39]
Stem bark and fruit	Maceration and cooking	Back pain, vision problems, anti-inflammatory and healing	[40]
Fruit	Tea (decoction), tea (maceration), maceration in a bath	Sore throat, hoarseness, leg pain, toothache, uterine inflammation, wounds, anemia, gastritis	[41]
Fruit, bark, roots and seed	Tea and tincture	Asthma, bronchitis, flu, fever, sore throat, sinusitis, diarrhea, rheumatism, blood clearance, kidneys and soothing	[42]
Fruits	Tea	Urinary infection	[43]
Fruits	Bottles	Infection	[44]
Stem bark, bast, fruits and seeds	Tea, “lambedor”, *in natura* and in powder	Infectious diseases, parasitic, circulatory, immune, cardiovascular, digestive, respiratory, genitourinary, musculoskeletal, conjunctive, injuries and poisoning	[45]
Seeds	Tea and immersed in water	Skin cuts, cough, flu and depression	[3]
Bark, fruit, and seeds	Decoction, infusion, maceration and syrup	Syphilis, cancer, depurative, diabetes, asthma, gastritis, bronchitis, sinusitis, stomach ache, rheumatism, sexual impotence, healing, bone fracture, headache, fever and throat infection	[46]
Whole plant and fruits	Infusion and maceration	Spine, blow, inflammation and kidneys	[47]
Leafs	Tea	All kinds of infection and inflammation	[5]

**Table 3:** Chemical classes or constituents found in *C. ferrea* extracts.

Part Used	Solvent	Analytical Technique	Constituents	Citations
Leafs	Cyclohexane	CG/MS	Octacosane, docosane, and heptacosan	[8]
Leafs	Water	HPLC	Ellagic acid and gallic	[7]
Leafs	Water at 25 and 100 °C	HPLC-DAD	Gallic acid, caffeic and ellagic epicatechin, quercetin, and luteolin and catechin	[48]
Leafs	Ethanol at 70%	HPLC	Ellagic acid and gallic, orientin and isovitexin	[49]
Leafs	Ethanol at 70%	NMR 1D e 2D	Gallic acid, brevifolin carboxylic acid, and brevifolin	[50]
Barks and seeds	Ethanol at 70%	HPLC	Ellagic acid	[51]
Barks	Water at 25 and 100 °C	HPLC-DAD	Gallic, caffeic and ellagic acids, catechin, epicatechin and quercetin	[48]
Barks	Water	RP-HPLC	Ellagic acid and gallic	[52]
Barks	Ethanol and water	LC-MS/MS	Kaempferol, quinolinic acid and gallic	[53]
Fruit	Ethanol at 96%	LC-HRMS/MS	Corilagin and ellagic acid and gallic	[1]
Fruit	Water, ethanol at 20–80%	HPLC-DAD	Ellagic acid and gallic	[7]
Fruit	Ethanol	HPLC	Ellagic acid and gallic	[54]
Fruit	Water	HPTLC e HPLC	Ellagic acid and gallic	[55]
Pods	N-hexane	GC-MS	N-dodecanol, myristic acid, methyl palmitate, palmitic acid	[56]
Pods	Chloroform	GC-MS	n-valeric acid, caproic acid, heptanoic acid, and octanoic acid	[56]
Pods	Ethyl acetate	GC-MS	Oxalic acid, butanedioic acid, pyrotartaric acid, and pentanoic acid	[56]
Pods	Alcohol at 70%	GC-MS	Glycerol, D-fructose, myo-inositol, and glucopyranose	[56]
Pods	Alcohol at 40%	HPLC-MS	Valonium dilactone acid, gallic acid derivatives, and ellagic acid	[12]
Pods	Ethanol and Water	LC-MS/MS	Ellagic acid, chlorogenic acid, and rutin	[54]
Pods	Water at 25 and 100 °C	HPLC-DAD	Ellagic acid and gallic, catechin, epicatechin, quercetin, and luteolin	[48]

GC-MS = thin-layer chromatography and Gas Chromatography-Mass Spectrometry; HPLC-DAD = High-Performance Liquid Chromatography with Diode Array Detection; RP-HPLC = Reverse Phase High-Performance Liquid Chromatography; HPLC = High Performance Liquid Chromatography; LC-HRMS/MS = Liquid Chromatography-High Resolution Tandem Mass Spectrometry; NaOH = Sodium hydroxide; LC-MS/MS = Liquid Chromatography Coupled to Tandem Mass Spectrometry; HPLC-MS = High-Performance Liquid Chromatography coupled to Mass Spectrometry; HPTLC = High-Performance Thin Layer Chromatography; NMR = Nuclear Magnetic Resonance.

**Table 4:** Bioactivities evaluated with different extracts of *C. ferrea*.

Parts Used/Solvents	Target or Model	Bioactivities Evaluated	Formulations/Dosage	Control (s)	Results	Citations
Full pod/Methanol	*Parvimonas micra* and *Porphyromonas gingivalis*	Antibacterial and anti-halitosis	In vitro 50–400 μg/mL for 72 h	Positive: chlorhexidine Negative: liquid medium	MIC: 50 and 120 µg/mL, respectively MBC: >50 and 130 µg/mL, respectively	[14]
Leafs/ Cyclohexane	*Bacillus subtilis*, *Escherichia coli*, *Proteus vulgaris*, *Pseudomonas aeruginosa,* and *Staphylococcus aureus*	Antibacterial	In vitro 0.04–25 mg/mL for 24 h	Positive: ampicillin Negative: DMSO 10%	MIC: 0.039, 0.039, 0.039, 0.39, 0.078 mg/mL, respectively	[8]
Leafs/Chloroform	*B. subtilis*, *E. coli*, *P. vulgaris*, *P. aeruginosa,* and *S. aureus*	Antibacterial	In vitro 0.04–25 mg/mL for 24 h	Positive: ampicillin Negative: DMSO 10%	MIC: 1.56, 6.25, 12.5, 3.12, 0.78 mg/mL, respectively	[8]
Leafs/Ethyl acetate	*B. subtilis*, *E. coli*, *P. vulgaris*, *P. aeruginosa,* and *S. aureus*	Antibacterial	In vitro 0.04–25 mg/mL for 24 h	Positive: ampicillin Negative: DMSO 10%	MIC: 0.78, 6.25, 12.5, 3.12, 1.56 mg/mL, respectively	[8]
Leafs/Methanol	*B. subtilis*, *E. coli*, *P. vulgaris*, *P. aeruginosa,* and *S. aureus*	Antibacterial	In vitro 0.04–25 mg/mL for 24 h	Positive: ampicillin Negative: DMSO	MIC: 6.25, 12.5, 25, 3.12, 3.12 mg/mL, respectively	[8]
Barks/Alcohol	*S. aureus* ATCC 10390, *P. aeruginosa* ATCC 9721, and *E. coli* ATCC 25,922 Wistar rats	Antibacterial and healing activity	In vitro 10 mg/mL for 24 h In vivo10 mg/mL for 28 days	Negative: bacterial nanocellulose membranes with extract	MIC: 0.39, 0.79 and 0.19 mg·mL^−1^, respectively	[67]
Fruits/Alcohol	*S. aureus*, *E. coli*, *Klebsiella pneumoniae,* and *P. aeruginosa*	Antibacterial	In vitro 20 μL of the crude extracts and in dilutions 1:2, 1:4, 1:8; 1:16 for 24 h	Negative: sterile water	Inhibition halos: 18, 12, 10 and 11 mm, respectively	[68]
Leafs and Fruits/Water	*Ralstonia solanacearum*	Antibacterial	In vitro 0.4–4.0 mg/mL for 24 h	Negative: water	70% inhibition at a concentration of 0.4 mg/mL	[69]
Pods and bark/Ethanol	*S. aureus*, *E. coli,* and *P. aeruginosa*	Antibacterial	In vitro 512–8 μg/mL for 24 h	Positive: amikacin, gentamicin, and clindamycin	MIC: 1024 µg/mL for all strains	[70]
Barks/Alcohol	*Staphylococcus* spp.	Antibacterial	In vitro Crude extract, 70% and 50% for 24 h	Positive: ampicillin, cephalexin, gentamicin, oxacillin, and penicillin Negative: saline	Inhibition halos: 61.1; 27.78 and 5.56% for the crude extract and concentrations of 70 and 50%, respectively	[71]
Leafs/ Propylene Glycol	*S. aureus* ATCC 6538	Antibacterial	In vitro Glycolic extract in concentrations of 3%, 5%, and 10% for 24 and 48 h	Negative: liquid soap	Average inhibition halo: 0.97 cm	[51]
Pods/Ethanol	*Streptococcus mutans*, *Streptococcus mitis*, *Streptococcus sanguis*, *Streptococcus sobrinus,* and *Lactobacillus casei*	Antibacterial	In vitro 0.97–500 mg/mL for 24 h	Positive: chlorhexidine gluconate	MIC: 15, 14, 14, 15, 15 mg/mL, respectively, and MICA: 31.2 mg/mL for all strains	[72]
Pods/Ethanol	*Staphylococcus aureus*, *Enterococcus faecalis*, *B. subtilis*, *E. coli*, *K. pneumoniae,* and *P. aeruginosa*	Antibacterial and antioxidant	In vitro 500–25 μg/mL for 24 h; 100–500 μg/mL for 30 min; 20 to 120 μg/mL for 10 min respectively	Positive: ascorbic acid and Trolox. Negative: specific medium	MIC: 125, 50, 50, 50, 125, 50 μg/mL, respectively; DPPH: EC_50_ 4.4 μg/mL and ABTS: EC_50_ 2.5 μg/mL	[73]
Pods/Alcohol	*Helicobacter pylori* Wistar rats	Antibacterial, antioxidant, antiulcerogenic and toxicity	In vitro 32–1024 μg/mL for 24 h In vivo 200 mg/kg for 14 days	Positive: amoxicillin, trolox, ranitidine, respectively. Negative: NaCl	MIC: 512 µg/mL; DPPH and ABTS: IC_50_ of 28.96 and 145.10 μg/mL, respectively; ED: 113 and 185.7 mg/kg; LD greater than 2000 mg/kg	[12]
Pods	Proteobacteria and Bacteroidetes	Antibiofilm	In vitro 0.5, 1, 2, 4, and 8 mg/mL for 48 h	Negative: sterile water	Inhibited growth by 82% at a concentration of 4 mg·mL^−1^	[74]
Seeds/ Ethanol	*Candida albicans* ATCC 10231, *Candida glabrata* CCT 0728, *Candida krusei* CCT 1517, and *Candida guilliermondii* CCT 1890	Antifungal	In vitro 4.8–5000 μg/mL for 48 h	Positive: ethanol 70%; amphotericin B and nystatin. Negative: specific medium	MIC: 9.7, 19.53, 78 and 39.06 µg/mL, respectively	[11]
Seeds/Ethanol	*C. albicans* ATCC 10231, *C. glabrata* CCT 0728, *C. krusei* CCT 1517, and *C. guilliermondii* CCT 1890; L929 fibroblast cells	Antifungal and Cytotoxicity	In vitro 7.81–1.000 μg/mL for 48 h	Positive: ethanol 70%	MIC: 9.7; 19; 78 and 4.8 µg/mL, respectively; toxicity at concentrations of 1000; 500 and e 250 µg/mL	[75]
Leafs/Water	*Colletotrichum* sp.	Antifungal	In vitro 0.156 mg/200 mL for 24 h	Positive: captan	Up to 96% inhibition at a concentration of 0.075 mg.mL^−1^	[76]
Leafs/Alcohol	*Colletotrichum* sp.	Antifungal	In vitro 0.156 mg/200 mL for 24 h	Positive: captan	100% inhibition of symptoms in treated seeds	[77]
Stem bark/Water, Ethanol and acetone	*Trichophyton rubrum* ATCC 28,189 and *Trichophyton mentagrophytes* ATCC 11481	Antifungal	In vitro 1.96–1000 mg/mL for 7 days	Positive: terbinafine Negative: DMSO	MIC: 62.5 and 31.3 μg/mL, respectively	[78]
---	*Lasiodiplodia theobromae*	Antifungal	In vitro 10, 20, and 30% for 5 days	Negative: sterile distilled water	Inhibited mycelial growth by 85.6% at a concentration of 30%	[79]
Leafs/Water	Wistar rats	Anti-inflammatory and antioxidant	In vitro 100, 200 and 300 mg for 24 h	Negative: saline 0.9% Positive: diclofenac 100 mg/kg	Effective doses: 100, 200 and 300 mg/kg;	[7]
Seeds/Water or Ethanol (20 - 80%)	Swiss mice and mouse embryonic fibroblast 3T3 cell line	Anti-inflammatory, antioxidant, antinociceptive, and cytotoxicity	In vitro 10, 15, 20, 25, and 30 µg/mL for 24, 48, and 72 h In vivo 50, 100, and 200 mg/kg for 20 and 30 min	Positive: diclofenac, cisplatin and ascorbic acid, and morphine Negative: saline	Effective doses: 50, 100 and 200 mg/kg;	[13]
Pods/Alcohol and ethyl acetate	ACP02 gastric adenocarcinoma cell line	Antioxidant and antimetastatic	In vitro 6.25 or 400 µg/mL for 20 min 6.25, 12.5, 25, 50, 100, 200, and 400 μg/mL for 24 and 48 h	Positive: doxorubicin Negative: medium RPMI	DPPH: IC_50_ 74.36 and 116.10 μg/mL ABTS: IC_50_ 9.76 and 29.13 μg/mL Decreased cell migration at concentrations of 50 µg/mL	[56]
Leafs/Ethanol	HaCaT and Wistar rats	Antioxidant, cytotoxicity, and hypolipidemic activity	In vitro 12.45 mg/L for 90 min; extract 50% for 45 min In vivo 300 mg/kg for 4 weeks	Positive: trolox, etoposide, lipanthyl, respectively	ED_50_: 12.5 µg/mL, IC_50_, 114.4 µg/mL	[50]
Leafs/Ethanol	Male Sprague–Dawley rats	Antioxidant, antihyperglycemic, and toxicity	In vitro 1 µg/mL for 30 min In vivo 250–500 mg/kg for 72 h and 1600, 2900, and 5000 mg/kg for 24 h	Positive: ascorbic acid Negative: normal rats	ED_50_: 12.45 µg/mL; reduced levels of liver function, serum glucose and a-amylase; non-toxic profile;	[9]
Leafs/Ethanol	---	Antioxidant	In vitro 0.39–100 μg/mL for 30 min	Positive: trolox	DPPH: IC_50_ 10.57 µg/mL e ABTS: IC_50_ 2.77 µg/mL	[10]
Leafs, branches and fruits/Ethanol and hexane	*Leishmania* (*Leishmania*) *amazonensis* and *Leishmania* (*Viannia*) *guyanensis*	Antileishmanial	In vitro 32–500 μg·mL^−1^ for 24, 48 and 72 h	Positive: pentamidine Negative: DMSO	Methanol extract from fruits and hexane from leaves: IC_50_ of 15.04 and 53.09 μg·mL^−1^*L.* (*L.*) *amazonensis*	[80]
Fruits/Ethanol	HT-29 e HEK-293	Antiproliferative, apoptotic and antioxidant	In vitro 12.5; 25; 50; 100 µg/mL for 24 and 48 h	Negative: untreated cells	Effective doses: 25–100 μg/mL	[54]
Barks and pods/Ethanol	B16F10 e NHF	Anti-wrinkle, anti-whitening and cytotoxicity	In vitro 0–250 μg/mL for 48 h	Negative: IBMX 25 μM	Effective doses: 25 and 250 μg/mL	[53]
Bark and seed/ethanol	Wistar rats	Acute toxicity maternal and fetal	In vivo 1.0; 2.5 and 5.0 g/kg for 14 days	Positive: 0.9% saline solution	Increase in creatinine levels in maternal serum and morphological changes in the fetus	[59]
Fruit/Ethanol	*Danio rerio* (Zebrafish)	Toxicity	In vivo 25, 50, 75, 125, 250, and 500 mg/L	Positive: water Negative: 1% propylene glycol	Concentrations of 25, 50, and 125 mg/L caused lethality in the embryos	[1]
Bark/Alcohol	Larvae of *Artemia salina* L.	Toxicity	In vitro 50, 100, 250, 500, 750, and 1000 µg/mL; 750 µg/mL for 24 h	Positive: sea water	CL_50_ of 822.6334 µg/mL	[81]
Fruit/Alcohol	Wistar rats	Toxicity and healing activity	In vivo *C. ferrea* 12.5 and 50% for 9 days	Positive: chlorhexidine digluconate Negative: NaCl 0.9%	Concentration of 12.5% exhibited epidermis constituted in all animals	[61]
Seed/Ethanol	*Astyanax* sp.	Genotoxicity	In vivo and In vitro 5, 10 and 20 mg/L for 96 h	Negative: not exposed	Increase of 2.5× in the level of DNA strands breaks in erythrocytes exposed to doses of 5, 10, and 20 mg/L	[82]
Leafs/Ethanol	HepG-2, Hep2, MCF-7, and HCT-116	Cytotoxicity	In vitro 5, 12.5, 25, and 50 μg/mL for 48 h	Positive: not exposed	IC_50_ of 19.3, 20, 21.8, and 24.47 μg/mL, respectively	[49]
Pods/Water	Meristematic roots cells of *Allium cepa*	Cytotoxic, genotoxic, and cytoprotective potential	In vitro 1 g/500 mL and 1 g/1000 mL for 24 and 48 h	Positive: water	Cytotoxic at concentrations 1 g/500 mL and 1 g/1000 mL after times 24 and 48 h of exposure	[83]
Pods	*Oryctolagus cuniculus*	Healing activity	In vivo Ointment in 16 and 24% for 21 days	Negative: glycerin and water	Ointment in 24% inhibited the lesion area	[84]
Stem barks/NaOH	Wistar rats	Healing activity	In vivo 0.025–0.1% for 21 days	Positive: collagenase 0.1 mL; negative: NaCl 0.9%	Effective concentrations: contractions 0.025, 0.05, 0.75, and 0.1%	[58]
Pods	Wistar rats	Healing activity	In vivo Ointment in 50% for 21 days	Positive: ointment collagenase	Significant reduction in the lesion area	[85]
Barks/Ethanol	---	Photoprotective activity and antioxidant	In vitro 0.005; 0.025; 0.050 e 0.100 mg/mL	Positive: Ascorbic acid	SPF of 3.29 in concentration 0.100 mg/mL and IC_50_ 27.53 µg/mL	[6]
Stem barks/Methanol	---	Arginase inhibitory activity	In vitro 10 μL for 30 min	Positive: Nor-NOHA	Inhibited 12.81% in the concentration 100 μg/mL	[51]
Seeds/water	Swiss mice	Inhibition of the hemorrhagic activity	In vitro Two venom to plant extract ratios 1:12 and 1:48 for 1 h	Positive: crude venom + saline Negative: crude venom + plant extract + saline	Showed no activity	[62]
Pods, bark and leafs/Methanol	Wistar rats	Edematogenic effect	In vivo 0.01, 0.1, 1 mg/Kg for 8 h	Negative: NaCl 0.9%	Effects at doses of 0.01–1 mg/kg	[86]
Stem barks/Water	Human third molars	Erosive potential	In vitro 50 mL tea + 0.1 mL 0.1 mol/L NaOH for 5 days	Positive: 1% citric acid	Loss of 37.03% dental enamel	[87]
Fruit barks	Flies of the *Calliphoridae* family	Repellent action	In vivo 20 and 50 % for 24 h	Positive: deteriorated bovine liver	Repellency of 97.5 and 100% in the concentrations 20 and 50%	[88]
Leafs and pods/Water and methanol	*Nasutitermes corniger* (Termite)	Insecticidal activity	In vitro 10, 25, 50, and 100 mg·mL^−1^ for 11 days	Negative: 0.1% Tween 80	Workers: CL_50_ 0.255–1.279 mg·mL^−1^ Soldiers: CL_50_ 0.146–8.003 mg·mL^−1^	[89]
Leafs/Alcohol	*Aphis craccivora* (Black aphid)	Insecticidal activity	In vivo 2.5 and 5 %	Positive: insecticide Negative: water	Efficiency of 51.71%	[90]
Leafs and pods/Water and methanol	*Dactylopius opuntiae* (Carmine cochineal)	Insecticidal activity	In vivo 200 mg/mL	Negative: 0.1% Tween 80	72.46–99.33% of mortality	[91]
Leafs and pods/Water and methanol	*Dactylopius opuntiae* (Carmine cochineal)	Insecticidal activity	In vivo 100, 50, 25, and 10 mg/mL for 10 days	Positive: chlorpyrifos, acetamiprid and thiamethoxam	Nymphs: CL_50_ 20–150 mg/mL Adults: CL_50_ 43–50 mg/mL	[92]
Leafs/Ethanol	*Alternaria alternata*	Control of brown spot of Alternaria	In vitro 100, 50, 25, and 10 mg/mL for 10 days for 12 days	Positive: cibenzolar-S-methyl Negative: water	Concentration of 500 μg/mL reduced in 52.0% the severity of disease	[93]
Leafs/Water and ethanol	*Alternaria alternata*	Control of brown spot of Alternaria	In vitro 1.0 mg/mL for 96 h	Positive: acibenzolar-S-methyl Negative: water	Concentration of 1 mg/mL reduced in 96.49 and 99.12% the severity of disease	[94]
Leafs	*Sorghum bicolor* L. (Sorghum)	Fertilizer	---	---	Increased the levels of potassium, calcium, and magnesium in the soil	[95]
Leafs and seeds/Ethanol	Seeds of *Cucumis melo* L.	Allelopathic potential	In vitro 1; 0.5; 0.25 and 0.125% for 8 days	Positive: water	30% abnormal seedlings at the concentration of 1%	[96]
Leafs, barks and roots/Water	*Calotropis procera* and *Cenchrus echinatus*	Allelopathic potential	In vitro Crude extract for 5 and 7 days	Negative: water	Inhibition of germination of both species	[48]
Dry leaves	*Vigna unguiculata*	Allelopathic potential	In vivo Proportion of sand: leaves 1:1/2; 1:1 e 1:2 for 70 days	Positive: water	Abnormalities in seedlings	[97]
Fruits	Meio aquoso contendo MB	Biosorbent	---	---	Fast kinetics and good adsorption in the removal of MB	[98]
Residues of pods	Captopril aqueous solutions	Biosorbent	Proportion of pod waste: ZnCl_2_ 0.5: 1; 1: 1 and 1.5:1	---	97.67% removal	[99]

MIC = Minimum Inhibitory Concentration; MBC = Minimum Bactericidal Concentration; MB = methylene blue; HT-29 = human colorectal cancer cell line; HEK-293 = embryonic renal cell line; NaOH = Sodium hydroxide; B16F10 = murine melanoma cell lines; NHF = normal human fibroblasts; HaCaT = keratinocyte cell line; IC_50_: Half Maximal Inhibitory Concentration; LC_50_: Median Lethal Concentration; ED_50_: Half Effective Maximum Dose; EC_50_: Half Maximal Effective Concentration; DPPH: 2,2-Diphenyl-1-picrylhydrazyl radical; ABTS: 2,2’-azino-bis (3-ethylbenzothiazoline); IBMX: 3-isobutyl-1-methylxanthine; RPMI: Roswell Park Memorial Institute (cell culture medium).

**Table 5:** Biological activities of compounds isolated from *C. ferrea* extracts.

Compound	Target or Model	Bioactivities Evaluated	Formulations/Dosage	Control(s)	Results	Citations
Galactomannan	Wistar rats	Antihyperglycemic and toxicity	In vivo10 mg/kg for 5 weeks	Positive: non-diabetic animals	Efficient dose of 10 mg/kg; No toxicity	[65]
Sulfated galactomannan	DENV-2 virus in Vero cells	Antiviral, antioxidant and cytotoxicity	In vitro 25, 50 and 100 μg/mL for 7 days	Positive: Vero cells infected DENV-2 Negative: Normal Vero Cells	96% inhibition against DENV-2 in the concentration of 25 g/mL; IC_50_ of 0.94 μg/mL	[100]
Brevifolin carboxylic acid	HaCaT	Antioxidant and cytotoxicity	In vitro 1–500 μg/mL for 72 h	Positive: Not exposed	ED_50_ 5 µg/mL and IC_50_ 124.9 μg/mL	[50]
2″-O-Galloylorientin	HaCaT	Antioxidant and cytotoxicity	In vitro 1–500 μg/mL for 72 h	Positive: Not exposed	ED_50_ 1.9 µg/mL and IC_50_ 67.5 μg/mL	[50]
2″-O-Galloylvitexin	HaCaT	Antioxidant and cytotoxicity	In vitro 1–500 μg/mL for 72 h	Positive: Not exposed	ED_50_ 3.8 µg/mL and IC_50_ 59.7 μg/mL	[50]
2″-O-Galloylvitexin	HepG-2, HCT-116, Hep2 and MCF-7	Cytotoxicity	In vitro 5, 12.5, 25, and 50 μg/mL for 48 h	Positive: Not exposed	IC_50_: 18.5; 22.6; 24.2 and 28.4 μg/mL, respectively	[49]

HaCaT = keratinocyte cell line; liver HepG-2, larynx Hep2, colon HCT-116, breast MCF-7 and prostate PC3, human cell line; ED_50_: Half Effective Maximum Dose; IC_50_: Half Maximal Inhibitory Concentration.
